# Molecular Mechanisms of Oxidative Stress in Acute Kidney Injury: Targeting the Loci by Resveratrol

**DOI:** 10.3390/ijms25010003

**Published:** 2023-12-19

**Authors:** Hina Rashid, Abdulmajeed Jali, Mohammad Suhail Akhter, Sayed Aliul Hasan Abdi

**Affiliations:** 1 Department of Pharmacology and Toxicology, College of Pharmacy, Jazan University, Jizan 45142, Saudi Arabia; 2 Department of Medical Laboratory Technology, College of Applied Medical Sciences, Jazan University, Jizan 45142, Saudi Arabia; 3 Department of Pharmacy, Faculty of Clinical Pharmacy, Al Baha University, Al Baha 65711, Saudi Arabia

**Keywords:** oxidative stress, reactive oxygen species, acute kidney injury, mitochondrial dysfunction, resveratrol

## Abstract

Reactive oxygen species are a group of cellular molecules that stand as double-edged swords, their good and bad being discriminated by a precise balance. Several metabolic reactions in the biological system generate these molecules that interact with cellular atoms to regulate functions ranging from cell homeostasis to cell death. A prooxidative state of the cell concomitant with decreased clearance of such molecules leads to oxidative stress, which contributes as a prime pathophysiological mechanism in various diseases including renal disorders, such as acute kidney injury. However, targeting the generation of oxidative stress in renal disorders by an antioxidant, resveratrol, is gaining considerable therapeutic importance and is known to improve the condition in preclinical studies. This review aims to discuss molecular mechanisms of oxidative stress in acute kidney injury and its amelioration by resveratrol. The major sources of data were PubMed and Google Scholar, with studies from the last five years primarily included, with significant earlier data also considered. Mitochondrial dysfunction, various enzymatic reactions, and protein misfolding are the major sources of reactive oxygen species in acute kidney injury, and interrupting these loci of generation or intersection with other cellular components by resveratrol can mitigate the severity of the condition.

## 1. Introduction

Generation of the free oxygen radicals is one of the most important physiological phenomena in the biological systems that are necessary for various vital functions. Free oxygen radicals were earlier regarded as toxic entities; however, over recent years they have gained recognition as necessary molecules for the growth and development of the organism [1]. The free oxygen radicals are biologically derived as byproducts of normal oxygen metabolism, chiefly in the mitochondria, while other respiratory pathways, cytochrome P450 (CYP450), and nitric oxide synthase (NOS) isoforms are also a source of reactive oxygen species (ROS) via numerous cellular pathways and most commonly include the peroxide, O_2_^•−^, singlet oxygen, ozone, hypochlorous acid, and hydroxyl radicals (OH^•^) and are collectively referred to by the umbrella term ROS [2,3,4,5,6]. ROS have an active role in physiological adaptations to hypoxia, cell proliferation and growth, vasoreactivity regulation, and signal transduction [7,8]. However, the amount of ROS generated is critical and needs to be maintained at a basal nontoxic level; majorly by the antioxidants present in the cell [9]. Any shift in this delicate balance and an increase in ROS production or decrease in free radical clearance leads to disrupted redox homeostasis, causing oxidative stress (OS) [10]. Oxidative stress is a well-identified critical factor in the emergence of numerous acute, chronic, and degenerative disorders that affect the majority of vital organs and systems and acts as a mediator in toxic responses [11,12,13]. The renal system is also a vital system that has ROS as an important mediator in disease and dysfunction. Numerous studies have shown an increase in oxidative stress and a notable disequilibrium in pro-oxidant and antioxidant activities in both humans and experimental animals with renal dysfunction [14,15,16,17]. Renal diseases remain an under-recognized health crisis, where the patient may be unaware of the condition due to a lack of symptoms at early stages, thereby preventing an early diagnosis [18,19]. However, before any clinical manifestation, molecular changes occur in the renal tissue and free radicals and prooxidants may be produced, which in turn can lead to a worse outcome of the disease [20]. The mitochondrial respiratory chain and reduced nicotinamide adenine dinucleotide phosphate (NADPH) oxidases in the kidney contribute to the prime sources of ROS. Since the kidneys have a large concentration of mitochondria and receive about 25% of the total blood supply, they are extremely susceptible to oxidative stress damage [21,22,23]. Most of the diseases involving renal impairments have elevated oxidative stress resulting from elevated oxidant activity and compromised antioxidant capacity, and the increase in oxidative stress is concomitant with increasing renal dysfunction [24]. Acute kidney disease, referred to as acute kidney injury (AKI), is one such disparate group of critical diseases that is marked by an abrupt renal function deterioration arising out of an abrupt drop in the glomerular filtration rate [25]. An increase in the incidence of AKI is observed the world over, with an estimated 13.3 million cases of AKI and 1.7 million global deaths per year [26]. AKI can result from trauma, surgery, sepsis, or nephrotoxic drugs, and has renal ischemia/reperfusion injury (IRI) as one of its leading causes. The condition is often preventable and treatable; however, in the absence of any treatment, there is a high risk of multiple organ failure and death [27]. The pathophysiological mechanisms of AKI comprise convoluted interactions among inflammatory, tubular, and vascular factors that are supervened by repair mechanisms, which may reinstate epithelial cells and normal renal function or lead to progressive fibrosis as well as chronic kidney disease [28,29]. oxidative stress represents one of the most important components in this pathological mechanism of AKI, being marked by circuitous and interdependent pathways leading to organ response as well as damage [30]. Therapies targeting ROS generation in such pathological conditions are currently being widely explored and most commonly include antioxidant enzymes from natural sources, synthetic agents with antioxidant activity, various antioxidant mimics, and vitamins. Resveratrol (RSV) is one such natural antioxidant that occurs in a wide array of plant species. RSV, a polyphenol, is produced by over 70 plant species in response to environmental stress such as mechanical injury, UV irradiation, and microbial infection [31]. The major food sources of RSV for humans are grape skin, blueberries, raspberries, mulberries, and peanuts, and it occurs in markedly high levels in grape juice and red wine [32]. Over the last few years, RSV has been the focus of numerous in vitro as well as in vivo studies that explore its pharmacological effects, including antioxidant, immunomodulatory, anti-inflammatory, hepatoprotective, antiatherosclerotic, anticancer, and antidiabetic properties [31]. While it modulates numerous mechanisms to confer protection, the organoprotective effects of RSV can majorly emanate from its antioxidative properties since RSV is an intensive scavenger of ROS and may thereby be used as a useful add-on therapy for preventing renal injury. Advantageous antioxidant attributes of RSV include direct scavenging of ROS, such as O_2_−, OH, and ONOO−, and modulation of the expression and activity of antioxidant enzymes, such as superoxide dismutase (SOD), catalase (CAT), and glutathione peroxidase (GPx), by regulating the transcription of nuclear factor E2-related factor 2 (Nrf2), forkhead box O (FOXO), activator proteins, or through enzymatic modification [33]. Patient-targeted studies of RSV are still in their infancy but numerous preclinical studies have strongly validated its therapeutic potential. This review aims to discuss various molecular mechanisms of oxidative stress in the pathology of AKI and the pharmacological targeting of such mechanisms by RSV.

## 2. Ischemia–Reperfusion-Induced Renal Injury

**Figure 1:**
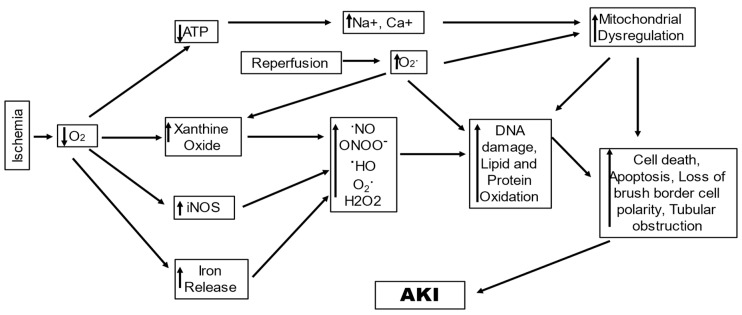
A schematic diagram showing ROS-generating mechanisms in ischemia/reperfusion-induced acute kidney.

IRI stands as a prevalent cause of AKI owing to poor oxygen and nutrition delivery and waste product clearance from renal cells. The demand and supply of oxygen and nutrients are not in balance, concomitant with waste accumulation that leads to tubular epithelial cell injury, and under severe conditions causes cell death by apoptosis and necrosis [34]. The underlying causes of AKI in IR-AKI are hypoxia and ischemia; they induce oxidative stress that consequently leads to tissue and function damage by disturbing microcirculation, cellular enzymes, and mitochondrial function (Figure 1) [35,36]. Initial changes reported with IR-AKI are the diminution of adenosine triphosphate (ATP) and alterations in the mitochondrial structure, which cause change and dysfunction in energy metabolism [37]. The number of mitochondria in the kidneys varies as per the cell type, which causes a difference in ROS production in various renal structures [35]. The ROS in mitochondria, referred to as mitochondrial ROS (mtROS), are generated at the complexes I and III of the electron transport chain (ETC) by the reaction of O_2_ with electrons that are derivatives of either nicotinamide adenine dinucleotide (NADH) or flavin adenine dinucleotide [38,39]. Under normal conditions of respiration, the mtROS are produced due to proton leakage; however, this is highly increased under conditions of stress [40]. In the IR-AKI, the surge in the ROS is known to potentially produce direct oxidative damage to mitochondrial lipids and proteins, which in turn disrupts mitochondrial bioenergetics via ETC function alteration and an increase in mitochondrial membrane permeability [41]. A study including in vivo exogenous and endogenous multiphoton imaging in the rodent model of induced nephrotoxic and ischemic AKI reported altered levels of mitochondrial NADH and proton motive force, with upregulated mitochondrial O_2_ levels and disjointed mitochondria, indicating mitochondria to be a prime source of ROS, and demonstrated the significance of alteration in mitochondrial function to be crucial in the onset of IRI-AKI. On the contrary, gentamycin exposure (AKI resulting out of toxic exposure) caused the first alterations in lysosomes in the renal epithelium, in addition to aberrance in brush border cells. This strongly indicates that mitochondria may be directly causative for increased levels of oxidative stress in AKI [42,43]. Moreover, mtROS are also known to lead to renal injury by invigorating the proinflammatory signals such as the nucleotide-binding domain, leucine-rich-containing family, pyrin-domain-containing-3 (NLRP3) inflammasome, and Toll-like receptors (TLRs) [44]. Jia et al., 2019, reported that renal ischemia resulted in oxidative stress injury and AKI by promoting mtROS generation via the downregulation of proliferator–activator receptor γ, which inhibited uncoupling protein 1(UCP1) expression. UCP1 located in renal tubular epithelial cells (RTEC) is downregulated during renal ischemic reperfusion in a time-dependent manner, while deletion of UCP1 leads to elevated oxidative stress in kidneys and exacerbates ischemia-induced AKI in mice [45].

Sirtuins comprise a family of signaling proteins attributed with an increasingly significant role in the regulation of cellular metabolism. Sirtuins belong to an ancient family of NAD+-dependent enzymes that are critical for the regulation of antioxidants and oxidative stress through the repair of DNA damage and metabolism via histone and protein deacetylation. Mammals feature seven sirtuins (SIRT1–7), found in the nucleus (SIRT1, SIRT6, and SIRT7), cytoplasm (SIRT2), and mitochondria (SIRT3, SIRT4, and SIRT5). SIRT proteins are gaining recognition in various diseases and dysfunction of the cell and are highly implicated in IR-AKI [46]. Diminished levels of SIR1 and SIRT3 in ischemia/hypoxia are reported to lead to decreased intracellular levels of nicotinamide adenine dinucleotide (NAD). This results from mitochondrial permeability transition pore (mPTP) opening, downregulation of a regulator of the NAD pool (nicotinamide phosphoribosyl transferase), and overactivation of poly (ADP-ribose) polymerases-1, resulting in a lowered activation of SIRT and consequent decreased SIRT1 levels. This decreased deacetylation function of SIRT1 causes its downstream targets to become acetylated, and inactive peroxisome-proliferator-activated receptor gamma coactivator 1-alpha elevates the level of oxidative stress by inhibiting manganese superoxide dismutase (MnSOD), promoting ROS generation, and lowering fatty acid β-oxidation [47]. Depleted SIRT3 is known to involve mitochondrial integrity, ATP production, and antioxidant defense system and increases oxidative stress, inflammation, and cellular apoptosis [46]. 

In tissues with ischemic injury, reperfusion is essential for survival; however, reperfusion itself leads to cell injury, known as reperfusion-injury, resulting from the generation of ROS [48]. This occurs due to the generation of hydrogen peroxide (H_2_O_2_) and superoxide (O_2_^•−^) during the conversion of accumulated hypoxanthine to xanthine in reperfusion. H_2_O_2_ forms highly reactive OH^•^ in the presence of iron; concurrently, NOS is induced in tubule cells by ischemia, which interacts with O_2_^•−^ to form peroxy nitrite, which causes oxidant injury as well as protein nitrosylation, leading to cell damage [49]. IR-AKI reperfusion leads to elevated ROS production when oxygen is reintroduced into mitochondria with ischemic injury. This increase in ROS may cause mitochondrial DNA (mtDNA) breaks, resulting in respiratory enzymes having mutant mtDNA-encoded defective protein subunits, which subsequently enhance impaired ATP and ROS production. This increase in ROS may also lead to tissue damage via cytochrome C release from the mitochondria initiating apoptosis or activating the immune response by other damage-associated molecular patterns (DAMPs) [50]. In the kidney, the proximal tubule seems to be specifically susceptible to the reperfusion injury mediated by ROS [51].

Wu et al., 2021, reported that renal tubular transient receptor potential ankyrin 1 (TRPA1) may act as an oxidative stress sensor having crucial regulatory functions in activating the signaling pathways and promoting the regulation of the transcription of IL-8. TRPA1 is a Ca^2+^ permeable ion channel that is ROS-sensitive, and hypoxia–reoxygenation injury caused an ROS-dependent TRPA1 activation. This caused an increase in intracellular Ca^2+^ levels, and activity of NADPH oxidase, Interleukin 8 (IL-8), in addition to activating mitogen-activated protein kinases/NF-κB signaling. These events were observed in the patients with AKI as well as in the mice with IRI [52]. 

Xu et al. (2020) also reported a significant role of MicroRNA-195-5p (miR-195-5p) as an oxidative stress modulator in IR-AKI, where expression of miR-195-5p was significantly lowered in I/R rat models, AKI patients, and hypoxia-incubated normal rat kidney epithelial (NRK-52E) cells. They also observed that treatment with an miR-195-5p mimic in rats reduced IRI-induced malondialdehyde (MDA) and reduced SOD oxidative stress by activating Nrf2/antioxidant response element in AKI [53].

Another mechanism that has gained considerable acknowledgment as a key mechanism of IR-AKI and involves ROS is ferroptosis [54]. It is an iron-dependent oxidative nonapoptotic cell death caused by ROS generated during the Fenton reaction, causing subsequent lipid peroxidation (LPO). The reaction of H_2_O_2_ with Fe^2+^ produces OH^•−^ with intense oxidizing properties; this reaction is called the Fenton reaction [55]. Ferroptosis is marked by massive consumption of plasma membrane polyunsaturated fatty acids that leads to plasma membrane rupture resulting from a lack of GPx 4 antioxidant systems and a consequent cell death [56]. The prime mediators of ferroptosis are iron metabolism and LPO [57]. During circulation, iron is bound to transferrin in the form of ferric iron (Fe^3+^), which is transported into cells by the membrane protein transferrin receptor 1 and then localized to the endosome. Inside the endosomes, iron reductase converts Fe^3+^ to ferrous iron (Fe^2+^), and divalent metal transporter 1 regulates the release of Fe^2+^ into unstable iron pools in the cytoplasm. Ferritin, the primary protein complex for storing iron, contains ferritin light chain (FTL) and ferritin heavy chain (FTH). These proteins store excess iron in a redox inactive form, shielding cells from oxidative harm. The expression of FTH and FTL is mainly in the proximal tubules of the kidney, with the major expression of FTL in the cytoplasm and that of FTH in the cytoplasm and nucleus, indicating FTH to have an antioxidant or gene regulatory role in the nucleus [58,59,60]. FTH shows iron oxidase activity and catalyzes the conversion of the Fe^2+^ to Fe^3+^, causing iron deposition into the ferritin shell, hence lowering the levels of free iron [61,62]. Iron in the cytosol is bound to Poly(rC)-binding proteins 1 (PCBP1), an iron chaperone, and transferred to ferritin via a direct protein-to-protein interaction [63,64]. In addition, nuclear coactivator 4 (NCOA4), a selective autophagic cargo receptor, is responsible for binding to ferritin and mediates the breakdown of ferritin via lysosomes [65,66]. The integrative actions of NCOA4 and PCBP1 on ferritin cause the adaption of the cells to fluctuations in iron availability. An increase in the cellular iron loads causes the free Fe^2+^ to mediate the binding activity of PCBP1 to ferritin, until saturation, and with further increase in Fe^2+^ ions, the efficiency of ferritin loading is reduced and inhibits ferritin turnover. Ferroportin causes the excess iron to be shuttled out of the cells and can lead to the generation of ROS mediating ferroptosis via Fenton-like chemistry [67]. Ferroptosis is manifested by aggregation of iron and ROS, which inhibits the activities of system xc- and GPX4 by the reduction of cystine uptake, reduced glutathione (GSH) depletion, and release of arachidonic acid, and other molecules [68]. GSH constitutes the prime intracellular antioxidant, with its synthesis being cysteine-dependent, and an inhibition of the cystine/glutamate antiporter Xc stimulates cell death via ferroptosis [69,70]. Depleted GSH levels can cause iron-dependent build-up of ROS, chiefly lipid ROS, which are adequate to kill cells [71]. In addition, loss of GPX4 activity evokes ferroptosis since GPX4 is a GSH-dependent enzyme and reduces lipid hydroperoxides to corresponding alcohols. Direct genetic evidence was provided by Friedmann et al., 2014, where the knockout of Gpx4 led to cell death in the form of ferroptosis. They demonstrated a critical role for the GSH/Gpx4 axis in the prevention of lipid-oxidation-stimulated acute renal failure and associated death using inducible Gpx4(−/−) mice [72]. In an in vivo study by Linkermann et al., 2014, ferrostatin 1 or ferrostatin (16–86) administration 15 min before ischemia in mice with severe IR-AKI resulted in reduced renal tissue damage, serum creatinine, and urea in the mice 48 h after ischemia, indicating a crucial role of ferroptosis in the pathogenesis of IR-AKI [73]. In mice, Jiang et al., 2021, also demonstrated pachymic acid to have a protective effect on IR-AKI. Pachymic acid was observed to cause the inhibition of ferroptosis in the kidneys via direct or indirect activation of Nrf2, and upregulation of the expression of the downstream ferroptosis related proteins, GPX4, solute carrier family 7 members 11, and heme oxygenase 1 (HO-1) [74]. This strongly suggests ferroptosis to be a key mechanism with its inhibition having therapeutic significance. 

It can be inferred with competent supportive evidence that oxidative stress is a core part of the interwoven processes between organelles and cellular mechanisms that lead to IRI-induced AKI. The mitochondria are at the center stage, leading to an increase in the generation of ROS and inflammatory responses that mediate the amplification of the injury. While the generation of mtROS during mitochondrial respiration is a normal physiological phenomenon, ischemia leads to a Ca^2+^ overflow, owing to a rapid loss of ATP, and aggravates mtROS production, which directly damages the structure and function of the mitochondria. Oxidative damage to the membrane lipids and proteins leads to compromised membrane permeability by opening the MPTP and loss of mitochondrial membrane potential [75]. Such damaged mitochondria bearing disrupted matrix cristae and presenting mitochondrial permeability-mediated necrosis are critical to ischemic AKI [76]. There is a loss of essential proteins in IR-AKI including those of the ETC, leading to abnormal ETC, which leads to an increase in ROS generation, leading to loss of mitochondrial membrane polarization, loss of selective permeability, and loss of the ion gradient that powers ADP phosphorylation [77]. The mtROS also triggers the inflammatory mechanism by acting as ligands for receptors such as NLRP3 inflammasome and TLRs. The mtROS are a major type of DAMPs that induce renal inflammation, even though other mechanisms may also be speculated to cause an inflammatory response. The mechanisms that intercede to lower the mitochondrial oxidative stress, such as the sirtuins SIRT1 and SIRT3, are downregulated, leading to obturation of the mechanisms that alleviate oxidative stress. Mechanism of oxidation and cell death ferroptosis, which is independent of the mitochondria, also becomes intense. Ferroptosis leads to an iron-dependent accumulation of oxidatively damaged phospholipids induced by disruption of the glutathione-dependent antioxidant defenses, leading to unrestrained LPO and consequent cell death. All these mechanisms generating excessive ROS can be convoluted in their pathways, aggravating the oxidative stress in IRI- AKI, leading to worsened conditions and poor treatment outcomes.

## 3. Sepsis-Induced AKI (SAKI)

**Figure 2:**
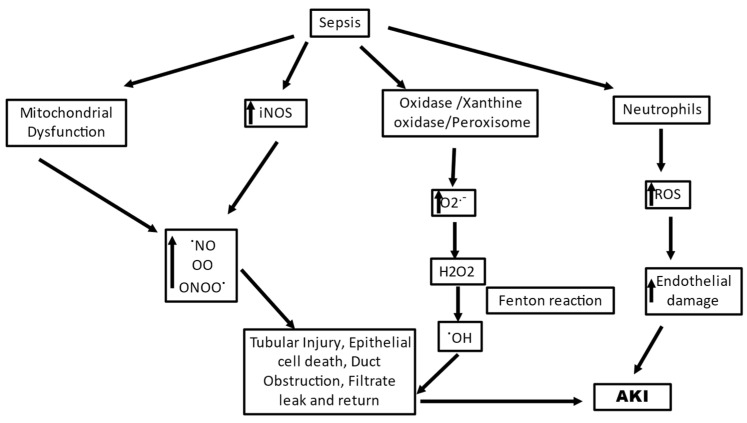
A schematic diagram showing ROS-generating mechanisms in sepsis induced acute kidney injury.

Sepsis is the most common cause of AKI in critically ill patients, accounting for 40–50% of cases and increasing the risk of in-hospital death six- to eightfold [78]. Sepsis is a condition characterized by progressive deterioration of systemic hemodynamics and systemic inflammatory response to infection. SAKI has both prerenal and renal events, in the environment of microvascular dysfunction, associated with its occurrence; however, the exact sequence of events still needs to be fully investigated [79,80]. Sepsis involves an immune disorder in the body and SAKI is manifested by varied regions of decreased peritubular blood flow and oxidative stress in the tubular epithelial cell [81]. Oxidative stress is an early significant event that, along with microvascular dysfunction, plays a pivotal role in the development of SAKI. A strong association between sluggish microvascular flow and oxidative stress in AKI has been well observed and a close coupling of oxidative stress with decreased blood flow in adjacent capillaries in renal tubules was reported [82]. Multiple-level ROS generation is seen in SAKI, and intense oxidative stress at systemic and intrarenal levels can lead to renal parenchymal damage directly and may intensify the dysregulation of microvasculature and function in renal tissue with a feedforward loop of hypoxia and ROS generation (Figure 2) [83]. A microbial invasion activates macrophages and neutrophils of the innate immune system, as the first line of defense which defends the host with oxidant molecules, such as O_2_^•− ^, OH^•^, peroxynitrite, and hypochlorous acid. The ROS such as H_2_O_2_ generated during the defense act as signaling molecules and can cause inflammation in nearby cells, leading to multiple organ failure [84]. Along with H_2_O_2_, neutrophils and macrophages have efficient enzymes to defeat invading pathogens; one such enzyme is myeloperoxidase. This enzyme catalyzes the conversion of H_2_O_2_ in the presence of chlorine ions to hypochlorous acid, which is a strong oxidant and microbicide agent. However, hypochlorous acid can react with host molecules and lead to pathological conditions as a side effect [85]. In SAKI, the most crucial role is played by dendritic cells and neutrophils in promoting renal disease [84]. The dendritic cells contribute indirectly to oxidative stress by recruiting and activating neutrophils, while the activated neutrophils can undertake phagocytosis or NETosis (formation of neutrophil extracellular traps) to neutralize the pathogens. Both the mechanisms are ROS-dependent mechanisms, where phagocytosis uses threat engulfment and “oxidative burst” activation, and NETosis requires NADPH oxidase 2 (NOX2) activation and O_2_ ^•−^ production [20,86]. A complete assembly and activation of NOX2 results in the formation of O_2_^•−^ via the transfer of electrons from cellular NADPH to molecular oxygen, and the activated neutrophils are recorded to produce almost 10 nmol/min O_2_^•−^ per million neutrophils during the oxidative burst [87]. In their study, Al-Harbi et al., 2019, reported that NOX2 blocking and induction of NO synthase in neutrophils decreased kidney injury in a mouse model of SAKI [84]. Zhuang et al., 2022, also reported the role of the NOX4 axis where NOX4-induced H_2_O_2_ plays a significant role in SAKI [88]. 

An inflammatory response in sepsis causes upregulation of inducible NO synthase (iNOS) leading to excessive generation of nitric oxide (NO), which leads to uncoupling of endothelial NO synthase (eNOS), in turn generating highly reactive O_2_^•−^s by oxidizing oxygen [89]. eNOS generates NO in two successive O_2_-dependent monooxygenation reactions of arginine that form L-citrulline. While the electron transfer in eNOS is a stringently regulated process, eNOS uncoupling can cause electron transfer to molecular oxygen other than to arginine and leads to the generation of O_2_^•^− [90]. This O_2_^•−^ generated by eNOS uncoupling is critically involved in various inflammatory conditions [87]. The NO that is excessively generated competes with SOD and reacts with O_2_^•−^ radicals, forming peroxynitrite protein adducts in tubules which can directly damage the tubular cells in SAKI [20]. Peroxynitrite is more cytotoxic than NO, being a strong oxidizing agent, and is involved in endothelial dysfunction [91]. The family of nitric-oxide-derived compounds, including nitroxyl anion, nitrosonium cation, higher oxides of nitrogen, S-nitrosothiols, and dinitrosyl iron complexes, form the reactive nitrogen species (RNS). They are significant for the functioning of numerous living cells; however, increased levels of RNS have been associated with nitrosative stress, causing cell injury and death [92]. Since there is a heterogenous expression of (iNOS), the varying concentrations of NO can cause uneven perfusion [93,94]. During sepsis, ROS and RNS generation can lead to activation of extracellular-matrix-degrading enzymes, damage to the cellular membranes, and inhibition of mitochondrial function, leading to damaged molecules in large amounts in organs and plasma, causing extensive oxidative and nitrosative stress. This causes a rapid shift from the initial capillary endothelial injury and renal hypoperfusion to a primarily oxidative stress-mediated tubular injury, which sustains the development of pathogenesis [93].

An undesirable stimulation of the pathogen-induced redox cycle of ROS originating from the mitochondrial respiratory chain is also a critical feature of SAKI. The mitochondrion is a primary source of signaling ROS, and any condition that leads to the production of large amounts of ROS transcending normal physiological levels can cause elevated mitochondrial dysfunction via a self-hyperbolizing ROS signal, which may have deleterious repercussions beyond the mitochondrion [20]. Renal mitochondrial injury is observed during the early stages of sepsis, concomitant with cellular damage arising out of ROS generation [95]. Mitochondrial damage is a critical source of cellular damage since the liberation of mitochondrial-damage-related patterns (such as mtDNA) can lead to activation of innate immune responses which can further escalate damage caused by oxidative stress [96]. It has been suggested by Chouchani et al., 2014, using comparative in vivo metabolomic analysis that characteristic metabolic pathways may be effective in mitochondria where O_2_^•−^ is generated through reverse electron transport at complex I of the ETC [97]. The mitochondria derive high-energy phosphate bonds of ATP by electrochemical proton gradient generated by the transfer of electrons via a series of electron carriers embedded in the mitochondrial membrane. This electronic transfer to molecular O_2_ is a highly regulated process that has only a small fraction (1-2%) of electrons leaking out in the process and reacting with O_2_, consequently forming O_2_− [98]. The primary loci of O_2_^•−^ production in the ETC are complex I and III; however, complex III is primal for O_2_^•−^ production in endothelial cells [99,100]. The O_2_^−^ generated in the mitochondria reacts with mnSOD in the mitochondrial matrix to produce H_2_O_2_, which in turn can cross the mitochondrial outer membrane and reach the cytosolic targets. The H_2_O_2_ assault on the cytosolic targets can cause numerous deleterious functional consequences such as stimulation of redox-sensitive transcription factors, proinflammatory cytokines, and inflammasomes [101,102,103]. mtROS can also contribute to increased oxidative stress through interactions with NOS [87]. A high metabolic burden observed during sepsis promotes an increased ROS production by mitochondria. Plotnikov et al., 2019, in their study reported altered structure and function of the mitochondria to be of prime importance in renal tissue damage in neonatal sepsis, and plastoquinol decylrhodamine 19, a mitochondria-targeted antioxidant, showed efficient nephroprotective action, indicating mtROS production as a critical pathway in SAKI [104]. Ge et al., 2017, in their study also suggested the involvement of miRNA-deregulated mRNAs in oxidative stress and mitochondrial dysfunction [105]. They reported that miR-4321 was involved in regulating the mammalian target of rapamycin (mTOR), NOX5, and Protein kinase B (AKT)1 expression, while miR-4277 was involved in regulating AKT3, Peroxisome proliferator-activated receptor gamma coactivator 1-alpha (PPARGC1A), Phosphatidylinositol 3-Kinase Catalytic Subunit Type 3 (PIK3C3), Wnt Family Member 1 (WNT1), and NOX5 expression, describing significant pathways of the predicted target genes related to oxidative stress and mitochondrial dysfunction. All these events have targeted impacts on mitochondria and redox homeostasis as a potential treatment strategy in sepsis-induced kidney injury since all these events have been documented for circumstances of systemic inflammation in the kidney [106].

To summarize, SAKI manifests the immune responses and microcirculatory impairments as the major source of the generation of ROS, characterizing an imbalance of the ROS and RNS. Excessive accumulation of O_2_^−^ is a hallmark of the condition that causes direct damage to cellular and vascular components. As soon as the inflammatory response is triggered in the body in sepsis, inflammatory mediators, including pathogen and damage-associated molecular patterns, are released into the intravascular area and are detected by TLRs on tubular and endothelial cells, which lead to further activation of these receptors, subsequently propagating a cascade contributing to tubular reparation, vascular rarefaction, and heightening of proinflammatory immune modulators at sites of injury, causing vascular congestion and endothelial dysfunction. All these processes merge to stimulate a superoxide-induced intensification of tissue hypoxia and cellular injury. These sepsis-induced vascular and tubular injuries, along with the oxidative stress, stimulate the engagement of polymorphonuclear neutrophils, leading to immunomodulatory events, causing a downstream production of ROS and RNS, which further aggravate the condition. The mitochondrial dysfunction, although not central, is also likely to have a complicated and significant contribution to the generation of ROS in sepsis. NO has a crucial role to play in this dysfunction, where it prevents the respiratory complex IV from functioning normally and increases the generation of O_2_^−^ while blocking normal electron transport and ATP synthesis by attaching to the particular complex. This generated O_2_^−^ combines with NO, again causing mitochondrial dysfunction, particularly through complex I inhibition.

## 4. Toxin-Induced Acute Kidney Injury (ToxAKI)

The kidneys are the main organs for maintaining homeostasis and are the primary eliminators of exogenous drugs and toxins, and in the course of this action, they can become the primary target of xenobiotic-induced toxicity [107]. The vulnerability of the kidney to these toxic effects is elevated because of its physiological features, which are manifested as the high flow of blood per gram tissue, the largest endothelial surface by weight, a dynamic system of diverse metabolizing enzymes, the presence of highly concentrated chemicals filtered in the tubular fluid adjacent to tubular cells, and unbinding proteins of various chemicals in the tubules, as well as intrarenal biotransformation reactions of chemicals. Most often, humans are unintentionally exposed to several classes of dissimilar toxicants that can have deleterious effects on the kidney and can lead to AKI [108]. These are broad-spectrum toxicants that include traditional and modern therapeutic agents (drugs), heavy metals, environmental and agricultural chemicals, toxic alcohol, drugs of abuse, animal venom, plant poisons, etc. [109]. Renal toxicity is caused by two main mechanisms: an excessive uptake of toxins by both the basolateral and apical transport systems, which damages the proximal tubular cells, and an excessive formation of tubular crystals as a result of the kidney’s concentrating ability. However, the underlying cellular pathophysiology in ToxAKI has oxidative stress as an important component [110]. 

Therapeutic agents such as antibiotics, chemotherapeutic agents, immunosuppressants, and analgesic agents have ascertained nephrotoxicity inflicted via different pathophysiological mechanisms. In the case of such drug-induced AKI, two modes of renal injury, acute interstitial nephritis (AIN) and acute tubular necrosis (ATN), are the prominently observed modes. AIN mostly arises as a result of drugs that instigate an allergic reaction, while ATN is an outcome of a direct toxic assault on tubular epithelial cells. However, at the cellular level, oxidative stress is one of the crucial underlying mechanisms that play an important role in the progression to ATN [111]. ATN is characterized by ROS generation arising out of the diminution or deactivation of GSH and other related antioxidants, consequently causing the accretion of endogenous ROS within the cells [112,113]. Misfolded proteins during protein folding in the endoplasmic reticulum can cause endoplasmic stress, which in turn can again initiate ROS production can lead to drug-induced AKI. Another highly liable mechanism of oxidative stress production during drug-induced AKI is the decoupling of oxidative phosphorylation and loss of mitochondrial membrane integrity from the respiratory chain, notably at Complex I and III [114]. O_2_^−^ is maybe the major deleterious oxidant produced by dysfunctional mitochondria instead of H_2_O_2_. The generation of ROS in the microsomes by the CYP450 enzymes is also a potent source of ROS induction in drug-induced renal injury [112]. Certain plants used in various traditional medicines can also lead to nephrotoxicity via oxidative stress by ROS generation and mitochondrial dysfunction [109]. 

The precise mechanism underlying nephrotoxicity caused by metal exposures is not yet fully determined; however, growing evidence suggests that metal-induced oxidative stress is a strong candidate for the same [115,116,117]. Being well recognized as catalysts in the oxidative reactions of biological macromolecules transition metals may exert their toxicities in various tissues due to induced oxidative stress [118]. The proximal tubule is the main site of metal-induced injury in addition to other glomeruli, vessels, and distal nephrons. In metal-induced nephron toxicity, exhaustion of intracellular GSH and free radical scavengers, antioxidant enzyme inhibition, and a significant increase in the levels of ROS are commonly manifested mechanisms. Metals like mercury, cadmium, and nickel cause the consumption of GSH while chromium, iron, copper, and vanadium undergo redox-cycling reactions. Fenton reaction is the major source of generation of the O_2_^•−^, and the OH^•^ with iron, chromium, vanadium, copper, and cobalt; however, arsenic is assumed to directly bind to the critical thiols [119,120,121]. Cadmium accumulates in mitochondria, blocking the respiratory chain at complex III, leading to increased production of free radicals that enhance caspase activity, causing renal damage [122,123]. Lead causes the induction of ROS by inducing the mitochondrial permeability transition, uncoupling of the respiratory chain, and intracellular depletion of GSH, cumulatively leading to increased oxidative stress and renal damage [124,125].

Agricultural and environmental chemicals are also known to cause AKI, especially in lower-economic countries. oxidative stress is a significant toxicological pathway of various pesticides such as organophosphates, bipyridyl herbicides, and organochlorines, probably due to their metabolism, mitochondrial disruption, or by hindering the antioxidant defense systems of cells [126,127]. These toxins can induce oxidative stress via cyclic oxidation/reduction, where one electron reduction by NADPH forms free radicals that donate their electron to O_2_, which produces the O_2_^•−^ radical. When NADPH is exhausted, O_2_^•−^ self-reacts to produce OH^•^ and leads to cell death [128]. Increased generation of ROS via mitochondrial dysfunction and accumulation of fatty acids in the cytosol, causing elevated fatty acid oxidation in peroxisomes and the endothelial reticulum, are also mechanisms observed in pesticide-induced AKI [129]. Environmental toxins such as aristolochic acid, melamine, and various industrial chemicals are also implicated in AKI via oxidative mechanisms [108,130,131].

This implies that AKI arising due to a toxic assault has oxidative stress as a critical component; however, the exact mechanism that leads to an increase in oxidative stress depends on the type of the toxin exposed, leading to a wide array of ROS-generating mechanisms. In the case of the development of drug-induced AKI, a defective antioxidant mechanism with depleted GSH stores is a major contributor to AKI, while endoplasmic stress, generated during protein folding in the endoplasmic reticulum, can also initiate ROS production, contributing significantly to AKI. Mitochondrial dysfunction comprising decoupling of oxidative phosphorylation and loss of mitochondrial membrane integrity primarily at Complex I and III leads to the generation of O_2_^−^ as a principal oxidant produced, which contributes to oxidative stress in drug-induced AKI. Microsomes have also been implicated in ROS generation in drug-induced renal injury. In case of exposure to occupational and environmental toxins, exhaustion of GSH stores, Fenton reaction, and mitochondrial dysfunction lead to ROS generation. The mitochondrial dysfunction in such toxins ranges from blocking the respiratory chain at complex III to altered mitochondrial permeability transition. An altered fatty acid metabolism comprising accumulation of fatty acids in the cytosol, leading to increased fatty acid oxidation in peroxisomes and the endothelial reticulum, is also a contributor of oxidative stress in pesticide-exposed AKI. This indicates a multifocal generation of oxidative stress in environmental toxin exposures, which presents a challenge to target, owing to a wide spectrum of included mechanisms and loci. 

## 5. Virus-Mediated Acute Kidney Injury

Viral infections are well documented to cause AKI. Viruses such as human immunodeficiency virus, hepatitis B virus, hepatitis C virus, hepatitis E virus, dengue virus, hantavirus parvovirus, SARS-CoV-19, cytomegalovirus, and Epstein–Barr virus infections, are manifested in AKI either by inducing glomerulopathy or glomerulosclerosis [132,133,134,135]. Viruses can cause AKI either directly via cytopathic effect or indirectly from consequent systemic and local responses of the immune system, both innate and adaptive [136]. The underlying cellular mechanisms, however, have oxidative stress as an element that inflicts pathogenicity. Numerous cellular mechanisms can lead to the formation of ROS in viral infections. During a host-virus interaction, ROS can be generated by biotransformation enzymes such as CYP450, xanthine oxidase, and spermine oxidase. Respiratory burst generating ROS as a defense mechanism can lead to increased oxidative stress having tissue-damaging effects. oxidative stress can result due to ROS production via the formation of HO-1 by the monocytes since monocyte production of HO-1 acts as a critical anti-inflammatory agent in regulating inordinate cell or tissue injury during oxidative stress and cytokinemia [137]. Virus infection can modulate the intracellular iron metabolism, triggering the Fenton reaction and subsequently producing OH^•^ [138]. Viral infections also mediate the accumulation of Ca^2+^ in the mitochondrial matrix, which leads to mtROS generation and cell death [139]. Protein oxidation in the endoplasmic reticulum causes ROS generation that causes oxidative stress [140]. ER and mitochondria must share signals which form very dynamical platforms known as mitochondria-associated membranes, and the ER-induced oxidative stress can lead to mitochondrial Ca^2+^ overload, leading to oxidative stress in virus infection [141,142]. Viruses have also been implicated in compromising the activity of antioxidants, both enzymatic (such as SOD, GPx, and CAT) and nonenzymatic (such as vitamin C, carotenoids, minerals, and cofactors) as a result of the inhibition of the expression of primary antioxidant enzymes brought on by the effects of viral regulatory proteins on cellular activity [143].

Briefly analyzing the abovementioned mechanisms leads us to conclude that while there is diversity in the pathogens causing AKI, the mechanism of pathogenesis does not miss out on oxidative stress as a significant factor arising out of numerous etiologies. Primarily host-virus interaction mediated by biotransformation enzymes can lead to ROS generation. Components of innate immunity, such as respiratory burst and monocytes, can contribute to oxidative stress. Fenton reaction can also be a significant contributor towards oxidative stress where the virus infection modulates the intracellular iron metabolism. Mitochondrial and endoplasmic reticulum are the organelles that are significant in generating ROS by the accumulation of Ca^2+^ in the mitochondrial matrix and protein oxidation, respectively. A decrease in the levels and activity of the enzymatic and nonenzymatic antioxidants is also a mechanism that promotes oxidative stress significantly in case of a viral infection. All these mechanisms are confirmatory of the multisite generation of oxidative stress in viral AKI, hence suggesting it to be a major contributor to pathogenesis. 

## 6. Resveratrol: Emerging as a Potential Antioxidant Nutraceutical

It can be inferred from the above-discussed oxidative stress-mediated mechanisms that ROS generation is not a causal but a consequential event in the pathogenesis of AKI. NAD(P)H oxidase, hypoxia, dysregulated mitochondrial pathways, and other cellular enzymatic reactions are critical in its development. Since ROS generation may be one of the many components in the development of AKI, a therapeutic agent that targets these ROS-generating mechanisms can be of specific benefit if the cause and course of the pathology are carefully evaluated. RSV stands as one of the highly studied polyphenols, gaining ground as a nutraceutical in the management of AKI with constituted antioxidant properties. 

The antioxidant properties of RSV emanate from scavenging diverse ROS and/or RNS, and numerous secondary organic radicals. The mechanism is the transfer of a hydrogen atom and successive proton loss. RSV also amplifies GSH levels, which are necessary for preserving the cellular redox equilibrium, in addition to increasing the expression of several antioxidant defense enzymes such as HI-1, CAT, GPx, and SOD [144]. These protective mechanisms are controlled by sirtuin 1, (Nrf2), and nuclear factor B signaling pathways [145,146,147].

RSV can be taken as an oral supplement, with a recommended dose of 250-1000 mg/day for up to 3 months [148]. Potential health benefits affiliated with RSV range from antimicrobial, neuroprotective, antiaging, anti-inflammatory, anticancer, cardioprotective, and blood-sugar-lowering properties, to even life-prolonging effects [149]. RSV is highly absorbed after oral consumption (75%) and then undergoes hepatic metabolism involving glucuronidation to form majorly resveratrol-4′-*O*-glucuronide or resveratrol-3-*O*-glucuronide, which is followed by the formation of resveratrol-4′-*O*-sulfate, resveratrol-3-*O*-sulfate, and resveratrol disulfates; however, it has a low bioavailability. Excretion is mainly through urine and feces [150,151,152]. The kidney has a high concentration of RSV, as observed in rats by oral administration of 3H-trans-resveratrol, leading to the highest observable levels in the liver and kidney [153]. Also, a single dose of trans-RSV (15 mg/kg body weight (b.w.) given intravenously in rats resulted in the highest levels in the kidney (1.45 nmol/g) and lungs (1.13 nmol/g) [154].

Numerous studies have been undertaken to evaluate the antioxidant activities of RSV in murine models of AKI. While a few studies have contradicted the preventive effects of RSV in AKI, the majority of the studies strongly validate RSV as a protective agent in AKI, with its antioxidant properties as mechanistically significant attributes [155,156,157,158]. Direct scavenging of ROS by RSV is known, in addition to modulating the expression as well as the activity of antioxidant enzymes, such as SOD, GPx, and CAT, via the regulation of the transcription of the Nrf2, activator proteins, FOXO, and SP-1, or through enzymatic modifications [159,160,161,162].

Prevention of both IR-induced LPO and depletion of antioxidant enzymes in renal tissue was reported by Şener et al., 2006, in the renal I/R-treated rats with pretreatment with RSV [163]. Short-term treatment with RSV was also reported to alleviate I/R-induced LPO, decrease damage to the renal cortex and medulla, and decrease the mortality rate from 50% to 10% in ischemic rats [164].

NO has significant functions in the renal tissue and is crucial for controlling renal blood flow and glomerular filtration rate. Upregulation of NO has also been reported to be a significant mechanism by which RSV asserts its antioxidant effects in IR-AKI. During an ischemic episode, NO exerts protection to the tissue due to its vasodilatory action, and NO can react with O_2_^−^ radicals during the reoxygenation phase, precluding a further chain of reactions for additional production of ROS [165]. Various studies have reported stimulation of NO production during renal I/R by RSV, thereby imparting protection to renal tissues with improved NO levels, in addition to lowering LPO and restoring the depleted renal antioxidant enzyme levels [166,167,168]. Even in a study where IR developed in kidneys due to a vital organ surgery, RSV has been reported to have antioxidant effects via upregulation of NO. Hemsinli et al., 2021 demonstrated that RSV had a protective effect against I/R-related AKI, which was an event associated with ruptured abdominal aortic aneurysm surgery by lowering oxidative stress and apoptosis. In renal tissues exposed to I/R, GSH, CAT, and NO levels increased; with RSV therapy, MDA levels, the amount of apoptotic renal tubular cells, caspase-3 levels, and tubular necrosis scores decreased [169].

The upregulation of SIRT1 activity by RSV in IR-AKI has been ambiguously reported in the literature. In a rat model, Bienholz et al., 2017, reported no protection conferred by RSV on I/R-induced AKI for the short term, as indicated by no significant difference in various study parameters between the control and treatment groups [158]. However, Gong et al., 2020, reported activation of SIRT1 by RSV, which significantly ameliorated renal function in IR-AKI. An in vitro study by the same group with human kidney 2 (HK2) cells, treated with hypoxia and reoxygenation, also reported RSV to reduce apoptosis and the production of ROS, indicating a protective effect of RSV [170]. One of the regulators of the expression of mitochondrial antioxidant defense in cells is PGC-1α; it manages the levels of antioxidant enzymes in the cell and elevates the SOD, CAT, and GPx activities to protect cells from mitochondrial dysfunction [171]. Olmos et al., 2013, reported that SIRT1 deacetylates PGC-1α and FOXO3 antioxidant genes in response to oxidative stress [172]. The absence of PGC-1α is known to lower the antioxidant enzymes, and treatment with RSV increases the mRNA expression levels of PGC-1α and SIRT1 and protein levels, thereby regulating mitochondrial homeostasis, restoring mitochondrial respiratory capacity, and reversing the aggravation of cell injury [173].

A promising role in targeting ferroptosis in IR-AKI is also alluded to, since it is known that cytoplasmic high-mobility group box 1 (HMGB1), a damage-associated molecular pattern molecule, leads to an increased tubular ferroptosis during IR-AKI, and a HMGB1 nucleocytoplasmic translocation promotes renal inflammation and kidney injury after I/R insult. RSV is reported to inhibit tubular HMGB1 translocation during I/R injury, indicating a promising treatment option [174]. 

In a study by Li et al., 2018, in a rat kidney IR-AKI model and H_2_O_2_-induced NRK-52E cells, RSV showed renal protection by decreasing ROS-induced stress via regulating Nrf2/TLRs/NF-κB pathways. Nrf2 regulates the antioxidant cell defense by acting as a protective transcription factor in IRI. Kelch-like ECH-associated protein 1 (Keap1) maintains the Nrf2 at a low and steady level by binding Nrf2 in the cytoplasm, thereby having a significant role in the adaptive response. Their study reported an upregulation of the Nrf2 expression and Keap1 by IRI, and treatment with RSV promoted Nrf2 expression, indicating that IRI likely relates to the adaptive response, which is activated by RSV [175].

In the case of SAKI, increased oxidative stress leads to the dysfunction of the renal microcirculation, distinctly by RNS. RSV has been observed to cause renoprotection in SAKI by decrement of oxidative stress, notably by decreasing RNS in preclinical studies [176,177]. Holthoff et al., 2012, in their study of sepsis-induced AKI using cecal ligation and puncture (CLP) in mice, reported RSV to have renoprotective effects. RSV treatment was observed to have melioration of oxidant stress of tubule, improvement blood flow along capillaries adjacent to the stressed tubules, abatement of tubular protein nitration, lowered azotemia, and prevention of tubular pathology; these preventive effects resulted from restored renal microcirculation, peroxynitrite scavenging, and iNOS inhibition. These attributes potentiated increased survival in the test model [178].

In a study with CLP-induced sepsis in rats by Xu et al., 2016, extraction of the kidney tissue and isolation of the RTEC was performed to study survival time evaluation. Sepsis was observed to lower SIRT1/3 activity, increase levels of acetylated SOD2 and oxidative stress, and damage mitochondria in RTECs. One of the deacetylated targets of SIRT1/3 is SOD2, and RSV treatment effectively restored the activity of SIRT1/3, reduced levels of acetylated SOD2, alleviated oxidative stress and mitochondrial function of RTECs, and increased survival time [179].

In sepsis-induced pediatric AKI, RSV was observed to palliate sepsis-induced injury by inducing the activation of the Nrf2 signaling pathway, leading to its dissociation from Keap1 and binding to the antioxidant response element (ARE) within the promoter of its downstream genes, including uridine 5′-diphosphate-glucuronosyltransferase, NAD(P)H: quinone oxidoreductase-1 (NQO1), HO-1, glutamate cysteine ligase, glutathione S-transferase, and GPx. The majority of these genes translate to significant antioxidant enzymes. This activation was tested in vitro also, using HK2 cells, where RSV potentiated, in a dose-dependent manner, the expression of HO-1 and NQO1 [180].

The suppression of the NF-κB pathway by RSV is a mechanism seen to lower oxidative stress in lipopolysaccharide (LPS)-induced renal sepsis in rats. ROS and/or RNS generated in sepsis activate the NF-κB signaling pathway, causing upregulation of the target genes of the NF-B pathway like iNOS, arachidonate 5-lipoxygenase (LOX5), cyclooxygenase-2 (COX-2), xanthine oxidoreductase, arachidonate 12-lipoxygenase (LOX-12), and NADPH oxidase NOX2 (gp91phox), which further elevate ROS and or RNS production. Treatment with RSV confers protection by suppressing the activation of this redox-sensitive transcription factor [181,182].

No study reports modulating ferroptosis by RSV treatment in SAKI; however, such studies are reported for LPS-induced endotoxemia cardiomyocyte injury, indicating a possible similar mechanism for AKI. miR-149 has an inhibitory effect on LPS-induced ferroptosis in cardiomyocytes, while upregulation of HMGB1 upregulation reversed this inhibitory effect. RSV upregulated miR-149 and downregulated HMGB1, thereby inhibiting the ferroptosis pathway and meliorating cardiomyocyte injury in LPS-induced endotoxemia [183].

A superior regulation of mitochondrial quality control proteins (Dynamin-related protein 1, PTEN Induced Kinase 1, Parkin, Bcl-2 interacting protein 3, and PGC-1α) associated with oxidative stress management was seen with RSV treatment in an LPS-induced sepsis model of AKI, in particular considering the role of H_2_O_2,_ as reported by Sabry et al., 2022. [184]. The H_2_O_2_ level stands as one of the factors that is an important modulator of the renin-angiotensin system (RAS) as well as ATP. It has been reported that in diabetics, a large amount of H_2_O_2_ is released from the heart, which, along with reduced ATP, may conduce to the signaling pathways of the mitochondria biogenesis [185]. Also, the skeletal muscles are observed to have a Na^+^-K^+^ ATPase redox sensitivity, and H_2_O_2_ scavenging reduced H_2_O_2_-interceded inhibition of the Na^+^-K^+^ ATPase [186]. The Na^+^-K^+^ ATPase oxidation was seen to promote its sensitivity to degeneration by a proteolytic pathway in myocytes. Also, ROS participate in angiotensin-induced regulation of Na^+^-K^+^ ATPase in the renal proximal tubule cell line through oxidative modulation of the pump [187]. Sabry et al., 2022 reported the alteration of mitochondrial quality control proteins by controlling H_2_O_2_ levels, restoring the renal ATP levels, and boosting the recovery of the renal tubule. Moreover, lowered levels of H_2_O_2_ meliorated alterations of the intrarenal RAS and lowered the resistive index of the renal artery. All these effects were attenuated with RSV administration in the sepsis model of AKI in their study [184].

Protection against initial polymicrobial-sepsis-induced AKI via inhibition of endoplasmic reticulum stress-triggered NF-κB pathway by RSV was also reported, which can be entailed to indirectly reduce oxidative stress. RSV ameliorated the stress in the endoplasmic reticulum, led to the inhibition of the phosphorylation of inositol-requiring enzyme 1 and NF-κB in the kidney, and was suggested to protect against septic AKI when administered at the earliest after the onset of sepsis and notably improved the prognosis of sepsis [180].

An intense antioxidant effect of RSV in LPS-induced nephrotoxicity was also suggested to involve iron shuttle protein by Li et al., 2022, since the mobilization of iron from the plasma to the kidney could eliminate LPS-induced sepsis in LPS-induced nephrotoxicity when treated with RSV in their study [188].

RSV treatment has also been reported to lower the DNA damage caused by oxidative stress in kidney cells, in addition to lowering MDA levels, increasing levels of GSH, and increasing activities of SOD and GPX, suggesting that RSV treatment might mitigate the sepsis-triggered oxidative DNA damage in sepsis-related diseases through the prevention of oxidative DNA strand breaks and activation of the DNA repair processes [189].

Propitious antioxidant properties are reported for RSV in ToxAKI as well and are known to alleviate the adverse outcomes of toxic exposure. Numerous studies have suggested lowering of the LPO and increase in the antioxidant enzyme activity with RSV treatment, where exposures to biotoxins, pesticides, and pharmaceuticals were involved [190,191]. In a study of kidney injury of alcohol-aflatoxin-B1-induced hepatocellular carcinoma, interesting findings were reported by Rawat et al., 2020. They reported an SIRT1-independent manner of oxidative stress protection by RSV in the kidney during alcohol-aflatoxin-B1-induced hepatocellular carcinoma. SIRT1 is closely implicated in the normal physiology and pathological conditions of the renal system, and an elevated expression of SIRT1 may imply renal toxicity. Attaining a suppressed expression of SIRT1 might be a favorable milieu for the cell, indicating that RSV may have some functions dependent on SIRT1, while others may be independent of it [192]. Ochratoxin is a mycotoxin that is commonly found to contaminate a wide array of foods and is a well-known nephrotoxicant that causes toxicity via several pathways, the increasing of oxidative stress being one of them. It is known to exhaust cellular antioxidant levels during acute exposure, leading to increased oxidative stress and consequent renal injury. In human embryonic kidney cells, RSV meliorated ochratoxin A toxicity by lowering intracellular ROS and elevating the expression of oxoguanine glycosylase 1, decreasing Nrf2 mRNA expression, and increasing concentrations of GSH. They indicated a profound antioxidant property of RSV, functioning independently of the influence of Nrf2. RSV also has a positive effect on the mitochondrial proteins influencing the redox balance of the cell. Ochratoxin caused an increase in (Lon Peptidase 1, mitochondrial) LonP1 and decreased SIRT3 expression [193]. SIRT3 is a regulator of LonP1 expression, and enhanced LonP1 expression is induced by oxidative stress to battle the accumulation of faulty proteins [194]. RSV significantly increased the SIRT3 expression which exhibits an inverse with LonP1 expression. SIRT1 was also activated by phosphorylation by RSV, influencing many cellular mechanisms, such as cellular metabolism and DNA repair, thereby having a remarkable influence on the cellular stress response [195]. The sequence of events in the stress defense is mediated by SIRT1 and an increase in phosphorylation modulates this mediatory role, leading to consequent deacetylation of stress proteins, such as PGC-1α and tumor protein p53, leading to protection of the cell, its components, and cell survival [196].

RSV modulates the activity of the p66shc isoform of SHC-transforming protein 1 in nicotine-induced injury in renal proximal tubule cells (NRK52E) to impart protection that counteracts the oxidative injury by enhancing the activity of the p66shc promoter. While p66shc is popularly known as a pro-oxidant enzyme increasing oxidative stress, some studies have revealed the antioxidant activities of p66shc under certain circumstances. This mostly transpires as the Ser36 phosphorylated p66shc does not translocate to the mitochondria, which indicates its pro-oxidant function; however, it activates the ARE, and, consequently, a set of antioxidant genes that harbor the ARE in their promoter. RSV activates the ARE via Nrf2, which induces the HO-1 promoter and, hence, offers protection to the cell via the cytoplasmic p66shc/Nrf2/HO-1 axis [197].

Renal toxicity inflicted by pesticides is also alleviated by RSV in a mechanism involving mitigation of oxidative stress and increasing antioxidant capacity. Malathion administration in rats was seen to significantly increase MDA levels in the kidneys and also increased nitrite oxide levels in addition to significantly decreasing the total antioxidant capacity level. RSV treatment led to lowered kidney MDA levels and nitrite oxide levels, and significantly increased total antioxidant capacity level in a dose-dependent manner [198]. Similar results were seen for fipronil, a pesticide known to largely mediate its toxicity through its effects on antioxidant systems, where RSV caused a decrease in LPO and increased antioxidant defense of the renal system [199]. In fish kidney cells treated with chlorothalonil, it was observed that RSV relieved the toxicity of chlorothalonil exposure by reanimating the miR-15a/BCL2-A20 axis disorders and inhibiting the expression of Cytochrome P450 Family 1 family genes [200]. Taking into consideration that ROS generation and the dysregulation of the microRNA expression have significant cross-talks, and can modulate each other by triggering various signaling pathways, RSV has a significant role in mitigating the toxicity induced by chlorothalonil and can be considered as a rescuer molecule that may be used to extricate the renal system.

Isorhapontigenin (ISO), a derivative of RSV, caused a retrieval of the paraquat-induced AKI in paraquat-intoxicated rat RTEC (NRK-52E) by mitigating apoptosis and oxidative stress. Treatment with ISO reduced oxidative stress markers such as ROS, MDA levels, and leakage of lactate dehydrogenase, in addition to increasing the antioxidants SOD, Nrf2, and oxygenase-1. Furthermore, a significant upregulation in the expression of Toll-interacting protein (TOLLIP) and sex-determining region Y box 9 (SOX9) in NRK-52E cells and the renal cortex was observed. Overexpression of SOX9 heightened transcription of TOLLIP and lowered apoptosis and oxidative stress induced by paraquat, while SOX9 knockdown vitiated the preventative effects of ISO on NRK-52E cells against PQ toxicity [201].

Renal toxicity induced by therapeutic agents is alleviated by RSV, as shown by reduced oxidative stress in anticancer and other therapeutic-agent-induced renal toxicities. One of the widely used chemotherapeutic agents, cisplatin, has nephrotoxicity as one of the most significant dose-limiting factors during its administration, with oxidative stress as a significant toxic mechanism. Valentovic et al., 2014, reported RSV to alleviate cisplatin-induced renal cortical cytotoxicity by modifying oxidative stress [202]. Cisplatin modulates various convergent points between numerous factors that are regulators of oxidative stress, leading to cytotoxicity, prominently being SIRT1, p53 upregulated modulator of apoptosis-α, B-cell lymphoma-extra-large (Bcl-xL), the cytosolic/mitochondrial cytochrome *c* ratio, and active caspase-3. Cisplatin was observed to lower the expression of SIRT1, decrease Bcl-xL in mouse proximal tubular (MPT) cells, and block the cisplatin-derived release of cytochrome *c* [203]. RSV activated SIRT1 and significantly prevented cisplatin-induced lowering of Bcl-xL in MPT cells, indicating renoprotection by activating various direct and indirect pathways of combating oxidative stress [204].

The toxicities associated with doxorubicin (DOX) have also limited its clinical use, spurring a need for an adjunct molecule that can lower its toxicity [205]. DOX is a known nephrotoxicant causing decreased kidney weight and marked deterioration in renal function [206]. RSV has shown promising results in lowering the nephrotoxicity associated with DOX treatment with lowering oxidative stress as one of the rescue mechanisms. RSV lowered nephrotoxicity toxicity by decreasing oxidative stress by improving mitochondrial function by protecting against mitochondrial damage in both oxidative phosphorylation and glycolytic pathways and possibly via SIRT1-mediated pathway [207].

An outcome of RSV treatment on DOX via modulating ferroptosis is not yet reported for renoprotection to our best knowledge, but the mechanism is well elucidated for cardiotoxicity. Reduced cell survival capacity and increased accumulation of iron and LPO in H9c2 cells were seen with DOX treatment, and pretreatment with RSV and inhibitor of ferroptosis ferrostatin-1 (Fer-1) reversed these effects. RSV was reported to inhibit the overproduction of mtROS and upregulate the p62- Nrf2/HO-1 pathway, which plays a crucial role in the regulation of DOX-induced ferroptosis in cardiomyocytes. This indicates RSV to be an influential p62 activator that has sanative significance in preventing DOX-induced cardiotoxicity by modulating ferroptosis, and the mechanism may be extended to renal protection in part or in toto [208].

In acetaminophen-overdose-induced nephrotoxicity in rats, RSV along with quercetin efficaciously protected against AKI and reduced tissue levels of p53, blood urea, creatinine, biomarkers inflammation, and oxidative stress, and alleviated glomerular ultrastructural alterations [209].

In hospital-acquired AKI, radiocontrast-induced nephropathy (RIN) is a leading cause that can occasionally warrant dialysis and increase the rate of mortality. There are currently very few clinical approaches available to prevent RIN. In vitro experiments with human renal proximal tubule epithelial cell lines (HK-2 cells) were seen to support renoprotection conferred by RSV in RIN. RSV significantly alleviated ioxitalamate-induced cytotoxicity by various mechanisms, including assuage of oxidative stress. RSV curbed ioxitalamate-induced formation of 8-hydroxy-2′-deoxyguanosine (8-OHdG), a biomarker of DNA damage induced by oxidative stress, and decreased ROS generation induced by ioxitalamate without activation of SIRT1 and SIRT3; however, it reversed the downregulation in Bcl-2 and survivin expression, causing relief from oxidative stress [210].

To date, there are no studies reporting the antioxidant activity of RSV in viral AKI to the best of our knowledge; however, studies have confirmed RSV to lower viral loads in infected cells. In herpes simplex virus (HSV)-infected African green monkey kidney cells (Vero), RSV suppressed, within the nucleus, the activation of NF-κB and impaired expression of vital immediate–early, early, and late HSV genes and synthesis of viral DNA induced by HSV [211,212]. RSV had a potent antiviral activity also observed in African green monkey kidney cells (Vero E6) up to 48 h post-infection. RSV interfered with the viral replication cycle and extended antiviral activity up to approximately five replication cycles of SARS-CoV-2 in vitro [213], indicating a protective effect that may have antioxidant activity as a contributor at some possible niche.

While in vivo and in vitro data confirm the beneficial effects of RSV in AKI, clinical trials are warranted to confirm its potential benefits in humans. Major benefits in humans are documented in trials from patients suffering from cardiovascular diseases, cancer, neurological disorders, obesity, diabetes, and nonalcoholic fatty liver disease; however, the trials have not been very conclusive and indicate that the therapeutic efficacy of RSV varies, depending on various patient and disease-related factors. Also, clinical trials documenting the effect of resveratrol on AKI are lacking to date, to the best of our knowledge, and only a few focus on chronic kidney disease.

Ghanim et al., 2010, found that 6 weeks of supplementation with 200 mg of *P. cuspidatum* extract containing 40 mg of resveratrol had a suppressive effect on oxidative and inflammatory stress in healthy individuals as compared to those taking placebo, as was indicated with mononuclear cells from the resveratrol group having suppressed NFκB binding, decreased ROS generation, and decreased tumor necrosis factor-alpha (TNFα) and IL-6. The same group reported that RSV and muscadine polyphenols suppress the increase in oxidative stress, lipopolysaccharide and lipoprotein binding protein concentrations, and expression of Toll-like receptor 4, cluster of differentiation 14, IL-1β, and suppressor of cytokine signaling-3 in mononuclear cells after a high-fat, high-carbohydrate (HFHC) meal [214].

In patients of nonalcoholic fatty liver disease (NAFLD), Chachay et al., 2014, treated patients with 3000 mg resveratrol for 8 weeks, and in another study, Heebøll et al., 2014 treated patients with 1500 mg resveratrol for 6 months. Both the studies reported no difference in the levels of alanine transaminase (ALT) and aspartate aminotransferase (AST), the lipid profiles, or expression of genes related to NAFLD between the resveratrol and the placebo groups [215,216]. However, in the study by Faghihzadeh et al., 2014, RSV was beneficial to the patients when used along with lifestyle modifications, such as exercise and diet, and the patients reported lower levels of ALT and inflammatory factors such as IL-6 and NF-κB, and improved lipid profiles. These studies indicate that RSV may be most effective under certain conditions, especially lifestyle changes [217].

In diabetic patients, Bhatt et al., 2010, reported that RSV treatment for 3 months caused a decrease in HbA1c levels, systolic blood pressure, total cholesterol, and total protein, improving glycemic control, but no significant lowering of fasting blood glucose levels, although the basal levels decreased [218]. Thazhath et al., 2016, found resveratrol’s efficacy in improving glycemic control to be indeterminate as RSV did not affect glucagon-like peptide 1 (GLP-1) secretion, glycemic control, gastric emptying, body weight, or energy intake [219]. Brasnyó et al., 2011 also found no changes in GLP-1 levels in diabetes patients. However, they observed a significant decrease in insulin resistance and blood glucose and delayed glucose peaks after meals [220]. This could have been due to RSV-induced decrease in urinary ortho-tyrosine excretion, which is a biomarker of oxidative stress, suggesting that resveratrol may be possibly used as an adjuvant therapy for diabetes treatment alongside the main pharmacological intervention to improve treatment outcomes.

While there are only two clinical trials documenting the effects of RSV in humans with kidney disease, the results are not very convincing. In a randomized, double-blinded pilot study by Saldanha et al. (2016), RSV treatment of 500 mg/day for 4 weeks to nondialyzed chronic kidney disease caused no significant effects, and the levels of antioxidant and anti-inflammatory markers were the same in RSV and placebo-supplemented groups; also, the administration of RSV for 4 weeks had a low toxicity [221]. Another randomized, double-blinded study by Lin et al., in peritoneal dialysis (PD), had promising results. Treatment of low-dose (150 mg/day) or high-dose (450 mg/day) RSV for 12 weeks resulted in significant improvements in mean net ultrafiltration (UF) volume and rate. In addition, the levels of angiopoietin receptor (Tie-2) and thrombospondin-1 (Tsp-1) in the peritoneal dialysate effluent were increased with RSV treatment, indicating angiogenesis-ameliorating effects in PD patients and improved ultrafiltration kidney function [222]. This observation indicates that administration of a specific dose of RSV (450 or 500 mg/day) for a longer duration may be required to stimulate beneficial effects.

Even though RSV emerged as a promising agent to help ameliorate pathological conditions in various diseases, several controversies surround the molecule, chiefly arising out of its lack of an efficient translation of in vivo and in vitro effects in human subjects. This may be due to the molecule interacting with numerous targets in the host molecules and also because of the vast variation of the designs carried out in human studies [223]. A fervent interest in the molecule emerged as a consequence of the identification of the “French Paradox” which was later characterized as a cyclooxygenase inhibitor and anticancer properties [224]. However, many of its potential benefits are under a question mark, barring its antioxidant property which still stands strong. The first question arose about the poor systemic bioavailability of RSV; however, it was soon revealed that the serum levels of metabolites of RSV (3-*O*-glucuronide) may be considerably higher than the parent compound, and the mean plasma level of RSV may be increased by micronization or by the combination with other compounds [225,226]. Another controversial study worth mentioning is the super estrogenic activity of RSV, which could have beneficial effects on cardiovascular events in addition to cancer chemopreventive activity [227]. However, a dark side emerged instantly, the idea that it could trigger estrogen-sensitive cancers in females. This doubt was laid to rest by studies with MCF-7, T47D, LY2, and S30 mammary cancer cell lines, where RSV did not function as an estrogen [228].

SIRT1 activation was credited as a significant mechanism of protection conferred by RSV and Pacholec et al., 2010, with their isothermal calorimetry, plasmon resonance, and NMR-based studies depicting no direct binding of RSV with SIRT. However, hypothesizing an indirect activation of the SIRT1 could not be evaded, where it is possible that RSV could lead to an activation of AMPK which in turn may activate SIRT1 [229].

In diabetic patients, treatment with RSV was also seen to have antioxidant effects, but improvement in disease endpoints such as inflammatory status, glucose homeostasis, blood pressure, or hepatic lipid content was dose-dependent and was no different than placebo at higher doses. This again placed RSV as a debatable moiety [230,231].

All these observations in humans place the therapeutic efficacy of RSV in perplexity. This can be a result of its poly-pharmacologic actions with numerous targets that may become entangled in human physiology in real-life settings, leading to suboptimal results. This, however, does not categorically disqualify RSV as a beneficial nutraceutical with clinical usefulness. A successful translation of preclinical studies to humans can be made possible with better delivery methods, combination formulas, and concomitant lifestyle changes. Even though a wide range of antioxidants and nutraceuticals are available, resveratrol can still be a protective molecule of choice if administered with proper conditioning. An advantage that needs to be tapped is its multitarget mechanisms. Since most pathological conditions have multi-mechanistic etiologies, RSV can target many of them to cause a better treatment outcome and/or improved life quality. This review, therefore, signifies the further research required to reprogram RSV to achieve better clinical translation in AKI and other pathological conditions.

## 7. Conclusions

The potential of RSV as an antioxidant is gaining stronger ground day by day. It exerts its protective effects via numerous mechanisms, with alleviation of oxidative stress as a major mechanism of exerting cell protection. RSV acts at various loci of oxidative stress generation in the convoluted loops of ROS generation in AKI and directly or indirectly reduces the severity of ROS-generated damage to the cell. While many points, molecules, and pathways modulated by antioxidant effects of RSV may yet be explored, seemingly SIRT, the NF-κB pathway, and mitochondrial bioenergetics are majorly modulated by RSV to confer nephroprotection in AKI.

Many other molecules and pathways are, nevertheless, significant. Although most of the studies are carried out in animal models in vitro, the prime antioxidant attribute of RSV makes it necessary to explore its nephroprotective effects in humans. RSV can be recommended as a supportive treatment or self-medicative prophylactic, although with caution.

## Data Availability

No new data were created or analyzed in this study. Data sharing is not applicable to this article.
